# Individual patient data systematic review and meta-analysis of optic nerve sheath diameter ultrasonography for detecting raised intracranial pressure: protocol of the ONSD research group

**DOI:** 10.1186/2046-4053-2-62

**Published:** 2013-08-06

**Authors:** Julie Dubourg, Mahmoud Messerer, Dimitrios Karakitsos, Venkatakrishna Rajajee, Erik Antonsen, Etienne Javouhey, Alessandro Cammarata, Michael Cotton, Roy Thomas Daniel, Carmelo Denaro, Emmanuel Douzinas, Clément Dubost, Moncef Berhouma, Behrouz Kassai, Muriel Rabilloud, Antonino Gullo, Abderrhammane Hamlat, Gregorios Kouraklis, Giuseppe Mannanici, Keith Marill, Sybille Merceron, John Poularas, Giuseppe Ristagno, Vicki Noble, Sachita Shah, Heidi Kimberly, Gianluca Cammarata, Riccardo Moretti, Thomas Geeraerts

**Affiliations:** 1 Université Claude Bernard Lyon 1, 69003 Lyon, France; 2 Département de Neuroscience Clinique, Service de Neurochirurgie, Centre Hospitalier Universitaire Vaudois, Lausanne, Switzerland; 3 Department of Intensive Care, General State Hospital of Athens, 154 Mesogeion Avenue, 11527 Athens, Greece; 4 Departments of Neurosurgery and Neurology, University of Michigan Health System, 3552 Taubman Health Care Center, SPC 5338, 1500 East Medical Center Drive, Ann Arbor, MI 48109 USA; 5 Harvard Affiliated Emergency Medicine Residency, Brigham and Women’s Hospital/Massachusetts General Hospital, 75 Francis Street, Boston, MA 02115 USA; 6 Service de Réanimation Pédiatrique, Hôpital Femme Mère Enfant, Hospices Civils de Lyon, Université Claude Bernard Lyon 1, 69000 Lyon, France; 7 Medical School, University of Catania, Catania, Italy; 8 Service des Urgences, Centre Hospitalier Universitaire Vaudois, Lausanne, Switzerland; 9 Department of Anesthesia and Intensive Care, Cannizzaro Hospital, Catania, Italy; 10 Third Intensive Care Department, Evgenidion Hospital, 20 Papadiamantopoulou Street, 11528 Athens, Greece; 11 Anesthesiology and Intensive Care Unit, Begin military Hospital, 69 Avenue de Paris, 94163 Saint-Mandé, France; 12 Service de Neurochirurgie A, Hôpital Pierre Wertheimer, Hospices Civils de Lyon, F-69000 Lyon, France; 13 Inserm, CIC201, Service de Pharmacologie Clinique, EPICIME, GroupementHospitalierEst, Hospices Civils de Lyon, Université Claude Bernard Lyon 1, 59 Boulevard Pinel, F-69000 Lyon, France; 14 Département de Biostatistiques, Hospices Civils de Lyon, 162 Avenue Lacassagne, 69003 Lyon, France; 15 Department of Anesthesia and Intensive Care, School of Medicine, Catania University Hospital, Catania, Italy; 16 Service de Neurochirurgie, HôpitalPontchaillou, 35033 Rennes, France; 17 Second Department of Propedeutic Surgery, Faculty of Medicine, National and Kapodistrian University of Athens, and Laiko General Hospital, 11527 Athens, Greece; 18 Department of Emergency Medicine, Massachusetts General Hospital, 55 Fruit Street, Boston, MA 02114 USA; 19 Intensive Care Unit, CH Versailles, Site André Mignot, 177 rue de Versailles, 78150 Le Chesnay, France; 20 Department of Cardiovascular Research, IRCCS – Istituto di Ricerche Farmacologiche “Mario Negri”, Milan, Italy; 21 Department of Emergency Medicine, Harborview Medical Center, 325Ninth Avenue, Seattle, WA 98104-2499 USA; 22 Department of Anesthesia and Critical Care, Ospedale SS Antonio e Biagio e Cesare Arrigo, viaVenezia 16, 15100 Alessandria, Italy; 23 Pôle AnesthésieRéanimation, Centre Hospitalier Universitaire de Toulouse, Université Paul Sabatier, 31059 Toulouse, France

**Keywords:** Meta-analysis, Diagnostic accuracy, Optic nerve sheath diameter, Individual patient data

## Abstract

**Background:**

The purpose of the optic nerve sheath diameter (ONSD) research group project is to establish an individual patient-level database from high quality studies of ONSD ultrasonography for the detection of raised intracranial pressure (ICP), and to perform a systematic review and an individual patient data meta-analysis (IPDMA), which will provide a cutoff value to help physicians making decisions and encourage further research. Previous meta-analyses were able to assess the diagnostic accuracy of ONSD ultrasonography in detecting raised ICP but failed to determine a precise cutoff value. Thus, the ONSD research group was founded to synthesize data from several recent studies on the subject and to provide evidence on the diagnostic accuracy of ONSD ultrasonography in detecting raised ICP.

**Methods:**

This IPDMA will be conducted in different phases. First, we will systematically search for eligible studies. To be eligible, studies must have compared ONSD ultrasonography to invasive intracranial devices, the current reference standard for diagnosing raised ICP. Subsequently, we will assess the quality of studies included based on the QUADAS-2 tool, and then collect and validate individual patient data. The objectives of the primary analyses will be to assess the diagnostic accuracy of ONSD ultrasonography and to determine a precise cutoff value for detecting raised ICP. Secondly, we will construct a logistic regression model to assess whether patient and study characteristics influence diagnostic accuracy.

**Discussion:**

We believe that this IPD MA will provide the most reliable basis for the assessment of diagnostic accuracy of ONSD ultrasonography for detecting raised ICP and to provide a cutoff value. We also hope that the creation of the ONSD research group will encourage further study.

**Trial registration:**

PROSPERO registration number: CRD42012003072

## Background

### Introduction

Raised intracranial pressure (ICP) is a common life-threatening condition that can occur in multiple neurological or non-neurological settings. The ‘gold standard’ for diagnosing raised ICP is the use of intracranial devices [1,2]. However, this requires an invasive method that has multiple disadvantages, namely severe complications (infection, hemorrhage, malfunction) [3-5] and non-feasibility due to absence of available neurosurgical expertise or contraindications (coagulopathy, thrombocythemia) [6].

Several non-invasive methods have been developed in order to propose an alternative, such as neuroimaging and transcranial Doppler sonography. However, the accuracy of these methods in predicting ICP values appears to be limited [7-10].

Optic nerve sheath ultrasonography provides a very promising bedside tool for the detection of raised ICP. Since the optic nerve is a part of the central nervous system, it is surrounded by cerebrospinal fluid (CSF). Thus, if CSF circulation is not blocked, an increase in ICP will be transmitted through the subarachnoid space surrounding the optic nerve, within the nerve sheath, especially the retrobulbar segment [11].

### Rationale for an individual patient data meta-analysis

Individual patient data meta-analysis (IPDMA) is considered to be the least biased method and ‘gold standard’ for addressing questions that cannot be resolved by a single study [12,13]. Several individual studies have demonstrated that optic nerve sheath diameter (ONSD) ultrasonography provides good diagnostic accuracy in the detection of raised ICP. However these studies have limited statistical power to provide a definitive cutoff value of ONSD to predict ICP above 20 mmHg (the usual threshold for raised ICP) due to small sample size. The two current published meta-analyses [14,15] of aggregated data from published studies identified six such studies with relevant data providing evidence on the diagnostic accuracy of ONSD ultrasonography, but they did not allow any clear conclusions on a pinpoint cutoff value. Indeed authors of each individual study have tried to determine the ONSD threshold in millimeters above which ICP is superior or equal to 20 mmHg, defining raised ICP by constructing a receiver operator characteristic (ROC) curve. This threshold varies from 4.8 mm to 5.9 mm according to studies [14-16]. An IPDMA is required to define an accurate cutoff. The other main advantage of IPDMA compared to meta-analysis of aggregated data is the potential to undertake data checking and ensure appropriateness of analysis. The statistical power is increased owing to the incorporation of individual patient covariates and differences between studies. The interaction of these covariates accounts for a greater proportion of explained data than analysis of mean values for patient characteristics and study differences performing with aggregated data.

### Objectives

The overarching objective is:

1) To establish an individual patient-level database from high quality studies of ONSD ultrasonography in the detection of raised ICP. We will assess the diagnostic accuracy of this non-invasive tool and address several key points.

The primary analytic objectives are:

2) To determine the diagnostic accuracy of ONSD ultrasonography in the detection of raised ICP (> 20 mmHg). The diagnostic accuracy will be expressed as sensitivity, specificity, and positive and negative likelihood values, including a diagnostic odds ratio.

3) To define the cutoff value for ONSD ultrasonography in the detection of raised intracranial ICP (> 20 mmHg). This value will be obtained from patient-level data.

For objectives 2) and 3), analyses will be performed exclusively using studies that compare ultrasonography with the ‘gold standard’ measure of ICP, invasive intracranial devices.

The secondary objectives are:

4) To determine whether the diagnostic accuracy of ONSD ultrasonography varies according to patient characteristics (for example age, weight, initial diagnosis, medical treatment).

5) To determine whether the diagnostic accuracy of ONSD ultrasonography varies according to study characteristics (for example experience of sonographer, trademark of devices).

Furthermore, this IPD MA will allow analysis of subgroups. We do not expect to find any difference in diagnostic accuracy between patient characteristics. However, it is mandatory to explore all possibilities of variation in order to make strong conclusions.

## Methods

This study is exempt from an institutional review board and/or ethical oversight because it involves analysis of de-identified data that has already been collected for a separate purpose. Therefore, it will not be possible to trace data back to individual patients.

### Search strategy and study selection

Two authors (JD, MM) will search Medline using PubMed interface, Embase, Pascal Biomed, Google Scholar and the Cochrane database from inception to January 2013. We will use the same search strategy as for our previous review [14]. Both authors will also review reference lists of identified studies manually and scanned abstracts from recent conference proceedings (from 2005 to 2011). Finally, ongoing trials will be searched using ClinicalTrials.gov. No language restriction will be applied.

In order to remove any clearly inappropriate titles, both authors will scan all retrieved references. Hard copies of all remaining papers will then be obtained and read by both authors to remove any for which there is no possibility of eligibility. Studies will be eligible if they actually assessed the diagnostic accuracy of ONSD ultrasonography with intraparenchymal or intraventricular devices for ICP monitoring. Studies will be excluded if invasive intracranial devices were not of the ‘gold standard’. Differences regarding eligibility will be resolved by consensus and with the help of the senior author (TG).

### Quality assessment

We will use the QUADAS-2 tool [17] to assess the quality of the studies. Two authors (JD and MM) will independently assess the quality of each study. High quality and low quality studies will be distinguished and grouped. Four primary criteria will be used as in our previous systematic review [14]: 1) the presence of an independent blind comparison with the ‘gold standard’; 2) inclusion in the population studied of an appropriate spectrum of patients on whom the test would be applied in clinical practice; 3) an adequate description of ultrasonography of ONSD to allow reproducibility of the method; and 4) a short delay (< 1 hour) between the two tests. High quality studies will have to fulfill all four criteria. Studies that do not fulfill these criteria will be qualified as lower quality. If we find important differences with regards to study quality, subgroup analyses will be performed for each different overall quality.

### Data collection

We will approach all authors whose studies meet the inclusion criteria to inform them about the IPDMA project and invite them to share their data in this collaborative study. If they are inclined to participate, we will request from them the following data for individual patients: age; sex; height; weight; baseline systolic arterial pressure; baseline diastolic arterial pressure; diagnoses; Marshall score, if applicable; Glasgow Coma Scale (GCS); type (hyperosmolar therapy, invasive ventilation, sedation, neurosurgery) and dose of treatment before ICP and ONSD measurements; delay between computerized tomography (CT)scan and ONSD ultrasonography; existence of any episode of raised ICP before ONSD ultrasonography; type of ICP devices (intraparenchymal or intraventricular) used; trademark of ICP devices; name and degree of clinical experience of the sonographer; trademark of sonography; frequency of the sonography probe; number of ONSD measurements and for each measurement, the existence of blinded measures; delay between the two measures; ONSD measures (transverse and sagittal planes for both eyes); ICP value; and delay since previous ONSD measurements.

Raised ICP will be defined *a priori* by invasive measurement > 20 mmHg in adults (age > 18 years old).

We will also ask authors to examine the provisional study list to identify any additional studies that they may be aware of, in order to include any study that may have been missed by our search criteria or that has not been published.

Individual patient data will be sought for all included studies and entered into a single database. Study level data will then be added to individual patient records.

This raw dataset will be saved in its original format, then converted to a Stata format (StataCorp, College Station, TX, USA), since Stata will be the statistical software used for analysis, and then saved again. Statistical coding will be written for the initial setup. Each variable will then be renamed to a standard notation and given a standard label. Any variable that cannot be identified or is ambiguous will be documented and appropriate clarification sought from the original investigator.

### Data validation

We will keep original data on a secure server with a backup copy according to a pre-specified data security agreement policy. Two authors (JD and MM) will crosscheck data from studies against data found in published articles. Any inconsistency will be discussed with the original author and corrections will be made when necessary. Requirements for authorship will be according to the International Committee of Medical Journal Editors, and a representative of each study will be invited to be part of the steering committee before publication to discuss analysis and results.

### Statistical analysis

The data synthesis will be performed using methods recommended by the working group of the Cochrane Collaboration on systematic reviews of diagnostic test accuracy.

For each study, we will construct 2 × 2 tables comparing the dichotomized test result with the final ICP status. We will then calculate sensitivity and specificity, and plot the results in a ROC space. We will perform a ROC analysis and an area under the curve (AUC) on ONSD in detecting raised ICP. A mean AUC across studies will be estimated and weighted for the sample size of each study.

Additionally, individual patient’s data from all studies will be pooled and directly analysed for diagnostic accuracy. Several analyses will be performed: 1) building of the empirical ROC curve and estimation of the area under the ROC curve, irrespective of the original study; 2) building of ROC curves based on a logistic regression model with mixed effects, in which the model will allow the odds ratio of the diagnostic test to vary according to the study; 3) modeling of the ROC curve according to the characteristics of the patients and studies. The approach developed by Alonzo *et al*. [18] will be implemented using Stata software. This analysis will allow quantification of the adjusted effect of the different characteristics on the diagnostic accuracy of ONSD; and 4) point estimation and interval estimation of the optimal threshold of ONSD, taking into account the prevalence of raised ICP in the studied population and the preference to avoid false negative or false positive results. The method developed by Subtil *et al*. [19] will be used to estimate the threshold with its credibility interval.

To perform sensitivity analysis, the analysis will be redone by leaving out one study. For the same purpose, we will also exclude low quality studies (defined above) and the analysis will be redone. Other sensitivity analyses will be undertaken in case of subsequently identified factors that would influence conclusions.

## Discussion

Our study will use individual patient data for the assessment of diagnostic accuracy of ONSD ultrasonography in the detection of raised ICP and to determine a precise cutoff value. We believe that the findings of the ONSD research group will have an important implication for both clinical practice and research. This IPDMA will provide the most reliable cutoff value of ONSD ultrasonography in the detection of raised ICP. This cutoff will no doubt be the starting point for new studies on this promising tool. Indeed, this cutoff will enable physicians to commence a large-scale trial to validate and test the tool in their own settings. Above all, we hope that the creation of the ONSD research group may serve as a model for future studies or research in this field. In this group, physicians, statisticians and other researchers have elected to share raw data and develop a robust partnership to improve clinical findings.

## Abbreviations

AUC: area under the curve; CSF: cerebrospinal fluid; CT: computerized tomography; GCS: Glasgow Coma Scale; ICP: intracranial pressure; IPD MA: individual patient data meta-analysis; ONSD: optic nerve sheath diameter; ROC: receiver operator characteristic.

## Competing interests

The authors declare that they have no competing interests.

## Authors’ contributions

All listed authors contributed substantially to the design of this protocol. All authors read and approved the final manuscript.

## References

[B1] Guillaume J Janny P Continuous intracranial manometry; physiopathologic and clinical significance of the method Presse Med 1951 59 45 953 955 14864407

[B2] Lundberg N Continuous recording and control of ventricular fluid pressure in neurosurgical practice Acta Psychiatr Scand Suppl 1960 36 149 1 193 13764297

[B3] The Brain Trauma Foundation The American Association of Neurological Surgeons: the Joint Section on Neurotrauma and Critical Care: recommendations for intracranial pressure monitoring technology J Neurotrauma 2000 17 6–7 497 506 10937892 10.1089/neu.2000.17.497

[B4] Paramore CG Turner DA Relative risks of ventriculostomy infection and morbidity Acta Neurochir 1994 127 1–2 79 84 7942188 10.1007/BF01808552

[B5] Wilberger JE Jr Outcomes analysis: intracranial pressure monitoring Clin Neurosurg 1997 44 439 448 10080020

[B6] Rickert K Sinson G Intracranial pressure monitoring Oper Tech Gen Surg 2003 5 170 175 10.1016/S1524-153X(03)70010-4

[B7] Winkler F Kastenbauer S Yousry TA Maerz U Pfister HW Discrepancies between brain CT imaging and severely raised intracranial pressure proven by ventriculostomy in adults with pneumococcal meningitis J Neurol 2002 249 9 1292 1297 10.1007/s00415-002-0844-8 12242556

[B8] Hiler M Czosnyka M Hutchinson P Balestreri M Smielewski P Matta B Pickard JD Predictive value of initial computerized tomography scan, intracranial pressure, and state of autoregulation in patients with traumatic brain injury J Neurosurg 2006 104 5 731 737 10.3171/jns.2006.104.5.731 16703877

[B9] Hassler W Steinmetz H Gawlowski J Transcranial Doppler ultrasonography in raised intracranial pressure and in intracranial circulatory arrest J Neurosurg 1988 68 5 745 751 3282040

[B10] Schmidt B Czosnyka M Raabe A Yahya H Schwarze JJ Sackerer D Sander D Klingelhofer J Adaptive noninvasive assessment of intracranial pressure and cerebral autoregulation Stroke 2003 34 1 84 89 10.1161/01.STR.0000047849.01376.AE 12511755

[B11] Hansen HC Helmke K The subarachnoid space surrounding the optic nerves: an ultrasound study of the optic nerve sheath Surg Radiol Anat 1996 18 4 323 328 10.1007/BF01627611 8983112

[B12] Riley RD Simmonds MC Look MP Evidence synthesis combining individual patient data and aggregate data: a systematic review identified current practice and possible methods J Clin Epidemiol 2007 60 5 431 439 17419953 10.1016/j.jclinepi.2006.09.009

[B13] Stewart LA Parmar MK Meta-analysis of the literature or of individual patient data: is there a difference? Lancet 1993 341 8842 418 422 10.1016/0140-6736(93)93004-K 8094183

[B14] Dubourg J Javouhey E Geeraerts T Messerer M Kassai B Ultrasonography of optic nerve sheath diameter for detection of raised intracranial pressure: a systematic review and meta-analysis Intensive Care Med 2011 37 7 1059 1068 10.1007/s00134-011-2224-2 21505900

[B15] Moretti R Pizzi B Ultrasonography of the optic nerve in neurocritically ill patients Acta Anaesthesiol Scand 2011 55 6 644 652 10.1111/j.1399-6576.2011.02432.x 21463263

[B16] Messerer M Berhouma M Messerer R Dubourg J Interest of optic nerve sheath diameter ultrasonography in dectecting non-invasively raised intracranial pressure Neurochirurgie 2013 59 2 55 59 10.1016/j.neuchi.2013.02.001 23523218

[B17] Whiting PF Rutjes AW Westwood ME Mallett S Deeks JJ Reitsma JB Leeflang MM Sterne JA Bossuyt PM QUADAS-2: a revised tool for the quality assessment of diagnostic accuracy studies Ann Intern Med 2011 155 8 529 536 10.7326/0003-4819-155-8-201110180-00009 22007046

[B18] Alonzo TA Pepe MS Distribution-free ROC analysis using binary regression techniques Biostatistics 2002 3 3 421 432 10.1093/biostatistics/3.3.421 12933607

[B19] Subtil F Rabilloud M A Bayesian method to estimate the optimal threshold of a longitudinal biomarker Biom J 2010 52 3 333 347 10.1002/bimj.200900242 20533411

